# Perceptions and Professional Career Intentions of Senior Nursing Students in India: A Cross-Sectional E-Survey

**DOI:** 10.17533/udea.iee.v44n1e05

**Published:** 2026-03-28

**Authors:** Nipin Kalal, Suresh K Sharma, Hem Lata, Inbarasi A, Sonam Meena

**Affiliations:** 1 RN, M.Sc. Assistant Professor. Email: kalalnipin@gmail.com https://orcid.org/0000-0002-1392-1787 All India Institute Of Medical Sciences India kalalnipin@gmail.com; 2 Professor, Ph.D. Email: sk.aiims17@gmail.com https://orcid.org/0000-0003-1214-8865 All India Institute Of Medical Sciences India sk.aiims17@gmail.com; 3 RN, M.Sc. Nursing Tutor. Email: hemlata19.sadhanu@gmail.com. Corresponding author. https://orcid.org/0000-0003-0517-4512 All India Institute Of Medical Sciences India hemlata19.sadhanu@gmail.com; 4 RN, B.Sc. Email: inbasri224@gmail.com https://orcid.org/0009-0006-6763-8588 All India Institute Of Medical Sciences India inbasri224@gmail.com; 5 RN, B.Sc. Email: meenasonam2811@gmail.com https://orcid.org/0009-0005-3535-2094 All India Institute Of Medical Sciences India meenasonam2811@gmail.com; 6 College of Nursing, AIIMS Jodhpur, Rajasthan, India All India Institute Of Medical Sciences College of Nursing AIIMS Jodhpur Rajasthan India; 7 Department of Nursing, AIIMS Mangalagiri, Andhra Pradesh, India All India Institute Of Medical Sciences Department of Nursing AIIMS Mangalagiri Andhra Pradesh India

**Keywords:** perception, students, nursing, attitude of health personnel, cross-sectional studies, India., percepción, estudiantes de enfermería, actitud del personal de salud, estudios transversales, India., percepção, estudantes de enfermagem, atitude do pessoal de saúde, estudos transversais, India.

## Abstract

**Objective.:**

To assess the perceptions and professional career plans of senior student nurses studying in various nursing colleges in India.

**Methods.:**

A cross-sectional E-survey was conducted pan-India through a Google form, and a total of 456 responses were received after obtaining voluntary consent. Data were collected through a self-structured Perception rating scale and a Professional career plan performance assessment.

**Results.:**

The majority of participants were young, unmarried females from western India, primarily pursuing a B.Sc. Nursing by personal choice. Regarding perceptions of the nursing profession, only 39.9% of participants had a high perception towards the nursing profession. 28.9% of senior student nurses intend to work abroad after completing their program. While 78.9% wish to stay in the profession, 21.1% consider changing careers, primarily due to a lack of respect. Perception scores were significantly associated with age and gender, showing that younger students and females had high perception regarding the nursing profession. Regression analysis confirmed that age had a negative and gender had a positive effect on perception.

**Conclusion.:**

The study concludes that age and gender significantly influence nursing students’ perceptions of the profession. It is recommended to implement targeted educational and motivational programs to enhance nursing students’ perception.

## Resumen

**Objetivo.:**

Evaluar las percepciones y los planes profesionales de los estudiantes de enfermería de último año que cursan sus estudios en diversas facultades de enfermería de la India.

**Métodos.:**

Se realizó una encuesta electrónica transversal en toda la India a través de un formulario de Google, y se recibieron un total de 456 respuestas tras obtener el consentimiento voluntario. Los datos se recopilaron mediante una escala de valoración de percepciones autoestructurada y una evaluación del desarrollo de los planes profesionales.

**Resultados.:**

La mayoría de los participantes eran mujeres jóvenes solteras del oeste de la India, que cursaban principalmente una licenciatura en enfermería por elección personal. En cuanto a la percepción de la profesión de enfermería, solo el 39.9% de las participantes tenía una percepción positiva de la misma. El 28.9 % de las estudiantes de enfermería de último año tiene la intención de trabajar en el extranjero después de completar su programa. Mientras que el 78.9 % desea permanecer en la profesión, el 21.1% considera cambiar de carrera, principalmente debido a la falta de respeto por la profesión. Las puntuaciones de percepción de la profesión de enfermería fueron más altas en los estudiantes más jóvenes y en las mujeres

**Conclusión.:**

Las variables edad y el sexo influyen significativamente en la percepción que tienen de la profesión los estudiantes de enfermería. Se recomienda implementar programas educativos y motivacionales específicos para mejorar la percepción de los estudiantes de enfermería

## Resumo

**Objetivo.:**

Avaliar as percepções e os planos profissionais de estudantes do último ano de enfermagem matriculados em várias faculdades de enfermagem na Índia.

**Métodos.:**

Foi realizada uma pesquisa eletrônica transversal em toda a Índia por meio de um formulário do Google, e um total de 456 respostas foram recebidas após o consentimento voluntário. Os dados foram coletados por meio de uma escala de avaliação de percepções autoestruturada e uma avaliação do desenvolvimento dos planos profissionais.

**Resultados.:**

A maioria dos participantes eram mulheres jovens solteiras do oeste da Índia, que cursavam principalmente bacharelado em enfermagem por escolha pessoal. Em relação à percepção da profissão de enfermagem, apenas 39.9% das participantes tinham uma percepção positiva da mesma. 28.9% das estudantes de enfermagem do último ano pretendem trabalhar no exterior após concluir o curso. Enquanto 78.9% desejam permanecer na profissão, 21.1% consideram mudar de carreira, principalmente devido à falta de respeito pela profissão. As pontuações de percepção da profissão de enfermagem foram mais altas entre as estudantes mais jovens e as mulheres.

**Conclusão.:**

As variáveis idade e sexo influenciam significativamente a percepção que os estudantes de enfermagem têm da profissão. Recomenda-se a implementação de programas educacionais e motivacionais específicos para melhorar a percepção dos estudantes de enfermagem.

## Introduction

The healthcare system is an evolving landscape that offers holistic care to patients and their beneficiaries. Nurses balance competency and compassion with their commitment to diversity, but they still leave their profession for various reasons. The World Health Organisation (WHO) estimates that by 2030, there will be a shortage of approximately 4.5 million nurses and 0.31 million midwives.^1^ The status of nurses in India and Southeast Asian countries mirrors the global trends. India has a Nurse-to-Population ratio of 1.7 per 1000 people instead of 3 as per WHO recommendations.^2^ These disparities underscore the strategic investment in nursing. Further focusing on investing in nurses becomes imperative to achieve an efficient, effective, resilient and sustainable health system.^3^ According to the Lancet Report 2022, approximately 2.8 lakh nurses complete nursing courses each year, whether it is a diploma, graduation, or post-graduation in nursing.^4^ But still, the shortage of nurses has not been conquered yet. This shortage causes a crisis in the healthcare system, which needs to be curbed by retaining the new nurses in the profession.

Senior Nursing Students are on the threshold of entering the nursing workforce. Their views on the nursing profession and career intentions provide a valuable insight into the workforce trends, challenges and opportunities. Many factors influence the professional trajectory of nurses, including education, experience, exposure to clinical practice, mentorship, and perception of the work environment. Their perception and career plan offer a whole new aspect to the nursing profession’s future. A study conducted in Ethiopia highlighted that only 54.4 % of nurses are willing to stay in the nursing profession.^5^ Further, in addition, another study stated that about 81.1% had a positive attitude towards the nursing profession but perceived nursing as a poorly remunerated, unpopular profession with bias.^6^

Senior nursing students recognise their career perspectives as a critical and significant decision. Despite extensive research conducted in various settings across India, the targeted cohort consisted of budding nurses rather than senior nurses, ultimately failing to identify a fading dedication, devotion, and a shift in attention to other areas. Therefore, the present study was conducted to assess the senior student nurses’ perceptions and professional career intentions. These findings will help inform strategies for improving nursing education, professional development, and retention of skilled nurses within the country.

## Methods

Study design. 

The study employed a descriptive cross-sectional E-Survey design. The study variables included socio-demographic characteristics, perception toward the nursing profession, and career intentions.

Setting, Participants and Sample size

The study was conducted among senior nursing students enrolled in recognized institutes of nursing across India (PAN India). Senior nursing students includes final-year or internship phase of their respective nursing program (B.Sc. Nursing, M.Sc. Nursing, Post. Basic B.Sc. Nursing and General Nursing Midwifery course). The participation was voluntary and anonymous. Data was collected via Electronic Media (structured Google form) over 6 weeks from 15 November to 30 December 2023. A total of 456 responses were received with complete responses considered as sample size. 

Bias Control

To minimize bias, participation was anonymous and voluntary. The online survey design prevented interviewer influence, and responses were automatically recorded to reduce manual entry errors. 

**Instruments**

Instruments were self-structured questionnaires developed after review of literature. The instrument consists of three parts: a) Sociodemographic Profile, b) Perception rating scale regarding role transition and c) checklist regarding career intentions. Socio-demographic profile section (part a) includes the background characteristics of the participants such as age, gender, marital status, religion, educational program, family income, and region of residence. 

The perception rating scale (part b) initially consisted of 18 items coving domains of professional image, social recognition and ethical practice. It was reviewed by a panel of seven experts (Senior nursing faculty, public health specialties, and nurse administrations) for relevance, clarity and comprehensiveness. Based on their suggestions, five items were deleted and final 13 items consisting of both positive and negative statements were validated with Content Validity Index (CVI) of 0.89. The tool was pretested and found to be reliable (Cronbach’s alpha of 0.82). 

The perception scale (Part b) was 4-point Likert scale (1 = Strongly Disagree, 2 = Disagree, 3 = Agree, and 4 = Strongly Agree). Negative statements were reversed scored prior to analysis. The total perception score ranged from 13 to 52, with high scores reflecting a more positive perception towards nursing profession. To categorize the perception level, a median score (37) was considered as cut-off and categorized as high perception (≥ 37) and low perception (< 37). 

The checklist regarding career intentions (part c) consisted of closed and open-ended questions to collect the comprehensive understanding of their career intentions and motivation towards nursing profession. It took about 12-15 minutes to complete the survey. This ensured clear definition and consistent measurement of variables in line with STROBE criteria for observational studies.

Ethical considerations

Ethical approval was obtained from the Institutional Ethical Committee (AIIMS/IEC/2023/4323). Informed consent is obtained from the participants.

Data Analysis 

The data were analysed using descriptive and inferential statistics in SPSS version 26. Descriptive statistics such as frequency, percentage, mean, and standard deviation were used to summarize socio-demographic characteristics and perception scores. Non-parametric tests such as Mann-Whitney U and Kruskal-Wallis were used to assess associations, while Spearman’s correlation and simple linear regression identified predictors. A p-value < 0.05 was considered statistically significant, and all analyses followed STROBE reporting guidelines.

## Results

Table 1 represents the socio-demographic characteristics of the participants. A total of 456 participants returned the Google form. Most participants were female (84%) and single (91.2%). The participants age ranged from 18-37 years with mean age of 21.79 ± 3.04 years. More than three-fourths of the participants belonged to the Hindu religion, and among them, only 36.2% had any siblings or relatives in the nursing profession. More than half (67.1%) of the participants were studying in a B.Sc. Nursing Course, and about 65% of the participants were from the Western region of India, including Rajasthan and Gujarat states. About 67.5% chose the nursing course on their own choice.

**Table 1:** Socio-demographic Characteristics (*n*=456)

Variables	Frequency (%)
Age in Years 18-22 23-27 28-32 33-37	320 (70.17) 114 (25) 17 (3.72) 5 (1.09)
Gender Male Female	73 (16) 383 (84)
Marital Status Single Married	416 (91.2) 40 (8.8)
Religion Hindu Sikh Christian Muslim	401 (87.9) 8 (1.8) 24 (5.3) 23 (5)
Do you have any sibling/ relatives from nursing profession? No Yes Ongoing / completed course	280 (61.4) 165 (36.2) 11 (2.4)
Course of Nursing Study M.Sc. Nursing B.Sc. Nursing Post Basic. B.Sc. Nursing GNM	51 (11.2) 306 (67.1) 8 (1.8) 91 (20)
Region Northern Region Eastern Region Western Region Southern Region	142 (30.5) 08 (1.75) 279 (60) 27 (5.80)
Choice of course selection By Own choice By Parent’s choice Did not get medical stream Going abroad By chance	308 (67.5) 78 (17.1) 52 (11.4) 1 (0.2) 17 (3.7)
Place of residence Hostel/ P.G Home Relative’s Home	254 (55.7) 189 (44.1) 13 (2.9)
Family income/month (In Rupees)* <10 000 10 001 - 30 000 30 001 - 50 000 >50 000	92 (20.2) 154 (33.8) 102 (22.4) 108 (23.7)

*One US dollar = 83 Rupees

Regarding the perceptions of senior nursing students regarding the nursing profession, the descriptive analysis of the perception statements reveals that mean scores closer to 4 indicate a high perception of the nursing profession. The total perception score ranged from 18 to 52, with a mean total score of 35.8 ± 6.2 (maximum possible score = 52). Based on the median score of 37, about 182 (39.9%) participants had a high perception (≥37), while 274 (60.1%) participants had a low perception (<37).

Table 2 depicts that participants strongly agreed that nursing plays an important role in patient care and recovery (Mean = 3.44, SD = 0.74), and contributes to society through health education (Mean = 3.34, SD = 0.67), while also recognising it as a humanitarian profession (Mean = 3.17, SD = 0.72). The profession was seen as economically admirable (Mean = 3.08, SD = 0.62) and offering opportunities to work abroad (Mean = 2.95, SD = 0.69), suggesting a favourable perception of its economic and global potential. However, mixed perceptions emerged regarding the nature of the profession, with some agreeing that it is predominantly female (Mean = 2.89, SD = 0.72) and that nurses often spend time on non-nursing tasks (Mean = 3.11, SD = 0.86). 

**Table 2:** Senior Nursing Students Perception regarding Nursing Profession (*n*=456)

Item	Statement about nursing Profession	Strongly Disagree *n* (%)	Disagree *n* (%)	Agree *n* (%)	Strongly Agree *n* (%)	Mean ± SD
	Is a humanitarian profession	27 (8)	8 (1.8)	282 (61.8)	139 (30.5)	3.17±0.72
	Plays an important role in patient care and their recovery	21 (4.6)	7 (1.5)	179 (39.3)	249 (54.6)	3.44±0.74
	Contributes in society health by providing health education	15 (3.3)	9 (2)	239 (52.4)	193 (42.3)	3.34±0.67
	Is predominantly female profession	25 (5.5)	74 (16.2)	285 (62.5)	72 (15.8)	2.89±0.72
	Similar to the servants’ job and doesn’t consider as Equal to other disciplines	118 (25.9)	86 (18.9)	192 (42.1)	60 (13.2)	2.43±1.01
	Is economically an admirable job	13 (2.9)	32 (7.0)	317 (69.5)	94 (20.6)	3.08±0.62
	Has an easy access to go abroad	25 (5.5)	48 (10.5)	308 (67.5)	75 (16.4)	2.95±0.69
	Receive recognition from the community	96 (21.1)	127 (27.9)	212 (46.5)	21 (4.6)	2.35±0.86
	Does not require eligibility criteria	245 (53.7)	100 (21.9)	85 (18.6)	26 (5.7)	1.76±0.94
	Provides an opportunity to get personal and family growth	131 (28.7)	23 (5)	284 (62.3)	18 (3.9)	2.41±0.94
	Hard profession that does not receive enough appreciation	118 (25.9)	63 (13.8)	258 (56.6)	17 (3.7)	2.38±0.91
	Is a caring profession in which ethical standards of care are maintained	72 (15.8)	8 (1.8)	231 (50.7)	145 (31.8)	2.98±0.98
	Personnel spend most of their time in non-nursing care	40 (8.8)	29 (6.4)	227 (49.8)	160 (35.1)	3.11±0.86
	Total score (Mean±SD)	-	-	-	-	36.29± 2.99

Participants expressed moderate disagreement with the notion that nursing does not require eligibility criteria (Mean = 1.76, SD = 0.94), indicating awareness of the professional standards involved. On the other hand, concerns were evident about the lack of community recognition (Mean = 2.35, SD = 0.86), limited opportunities for personal and family growth (Mean = 2.41, SD = 0.94), and the perception that nursing is a hard profession that receives insufficient appreciation (Mean = 2.38, SD = 0.91). The idea that nursing is similar to a servant’s job and not equal to other disciplines received a mean score of 2.43 (SD = 1.01), reflecting divided opinions and some lingering stigma. Overall, while the profession is valued for its societal contributions and ethical foundations, perceptions indicate ongoing concerns regarding recognition, role clarity, and professional growth.

Table 3 indicates that the majority (71.1%) of senior student nurses intend to work in India after completing their program, while 28.9% prefer to work abroad. Most plan to join clinical institutes (42.5%), followed by teaching (30.4%) and nursing administration (17.3%). A large proportion (85.1%) wish to work in the government sector. Regarding higher education, 87.7% plan to pursue further studies, mainly aiming for M.Sc. Nursing (43.2%) and Ph.D. Nursing (23.9%). While 78.9% wish to stay in the profession, 21.1% consider changing careers, primarily due to a lack of respect (52%). Other reasons include disinterest, health issues, and social stigma.

**Table 3:** Professional Career Intentions of 456 Senior Nursing Students

Professional Career Intentions of Senior student Nurse’s				Frequency (%)
Intent to work after completion of program	India	324 (71.1)
Abroad	132 (28.9)
Plan to join after graduation/diploma	Teaching institute	139 (30.4)
	Clinical institute/Bedside nursing	194 (42.5)
	Nursing Administration	79 (17.3)
	Independent nurse practitioner	44 (9.6)
Desired sector to join after completion of the program	Government	388 (85.1)
	Semi-govt	30 (6.6)
	Private	38 (8.3)
Plan for higher education If yes which course,	Yes	400 (87.7)
	No	56 (12.3)
	
	Post-basic B.Sc. Nursing	64 (14%)
	M.Sc. in Nursing	197 (43.2)
	Ph.D. in nursing	109 (23.9)
	Nurse Practitioner	25 (5.5)
	M.Sc. in Philosophy	05 (1.1)
Plan to change the profession	Yes	96 (21.1)
	No	360 (78.9)
Reason to change the profession	Not Interested	19 (19.7)
	Lack of respect	50 (52.0)
	Health issue	14 (14.5)
	Inferiority complex	7 (7.29)
Social stigma	6 (6.25)

With regard to the association between Student nurses’ perception with selected demographic variables in Table 4 depicts the association between student nurses’ perception and selected demographic variables, a statistically significant relation was found between age and low perception (*p*=0.027), indicating that perception scores varied significantly across different age groups among those with low perception. Specifically, younger participants (aged 18-22 years and 23-27 years) had higher mean ranks compared to older age groups, suggesting that younger students were more likely to report lower perceptions. However, no significant association was observed between age and high perception (*p*=0.311), implying that age did not meaningfully influence the perception scores among students with high perception. Regarding gender, a significant difference was noted in the low perception group (*p*=0.004), with female students having a higher mean rank than male students, indicating that females were more likely to have higher perception scores even within the low perception category. Conversely, no significant association was found between gender and high perception (*p*=0.964), suggesting that gender did not play a differentiating role among those with high perception. Overall, the findings suggest that age and gender may influence perception levels, particularly among students with lower overall perceptions of the nursing profession.

**Table 4:** Association between level of student’ nurses’ perception with selected demographic variables

Demographic variables		**Low perception (*n*=274)**			High perception (n=182)
Mean Rank	***p-*value**	Mean Rank	***p-*value**
Age in years	18-22 23-27 28-32 33-37	143.3 134.3 87.4 64.2	0.027^$^	92.72 93.31 62.83 27.00	0.311
Gender	Male Female	108.86 143.89	0.004^#^	91.04 91.57	0.964

Note: ^$^ Kruskal Wallis test, ^#^Mann-Whitney test

*Correlation Analysis between perception score and selected demographic variables* shows a weak but statistically significant negative correlation between age and perception score (r=-0.142; *p*=0.001). Older individuals tend to have slightly lower perception scores, but the relationship is not strong. The scatter plot illustrates the relationship between student nurses’ perception scores and their age. The dotted red line represents the line of best fit, suggesting a weak negative correlation (R² = 0.020, *p*>0.05), indicating that as age increases, perception scores tend to slightly decrease.

**Figure 1:**
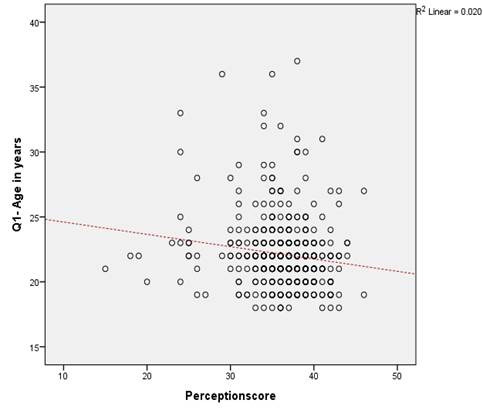
Correlation Analysis between perception score and selected demographic variables

In the linear regression, age and gender were significant at a *p*-value of 0.005. Overall model was statistically significant [(2, 453) = 8.67, *p*<0.001, and explained only 3.3% of the variance in perception scores (Adjusted R² = 0.033). Age was identified as a significant although weak negative predictor of perception (B = -0.88, SE = 0.31, β = -0.13, *p* = 0.005), indicating that as age increased, perception scores decreased. Whereas, gender was a positive and weak predictor (B = 1.41, SE = 0.52, β = 0.12, *p* = 0.007), suggesting that females had high perception scores than males by an average of 1.41 units, with a 95% confidence interval of 0.39 to 2.42.

## Discussion

The focus of this study was to explore the senior student nurses’ perceptions and professional career intentions while studying in a nursing college in India. The findings revealed that the majority of participants were female (84%) and single (91.2%), reflecting the traditionally female-dominated nature of the nursing profession in India. A significant portion of the sample (67.1%) was pursuing a B.Sc. Nursing primarily belonged to the western region of India. These demographic trends align with previous literature that highlights the concentration of nursing students in specific geographic and educational backgrounds.^7^^,^^8^ Further, more than half (67.5%) of senior student nurses chose nursing by their own choice; these results were consistent with the results where 81.8% of participants chose the nursing profession on their own.^9^

Senior Student nurses had having low perception (60.1%) towards the nursing profession. These results were inconsistent in setting countries and others, highlighting that one fourth of nurses only had a low perception regarding the nursing profession.^10^^,^^11^ Whereas, on stating the response on perception item, there were consistent results with our study results states that lack of community recognition (46.5%), insufficient appreciation (56.6%), limited opportunities for personal and family growth (62.3%), Humanitarian profession (61.8%), and plays an important role in patient care (54.6%), etc.^12^

This study also identifies, nearly three-fourths (71.1%) of senior student nurses intend to work in India after completion of work, desirably in the Government sector (85.1%). These results were similar to the other Indian study, where B.Sc. nursing students are less willing to go abroad. This can be due to wider career opportunities in India, particularly in the Government Sector^.(^^13^ Every year NORCET (Nursing Officer Recruitment Common Eligibility Test) examination is conducted, where nursing aspirants used to prepare during their nursing course it, Government salaries are better as compared to those in private institutions. Study results reflected that the majority of senior nursing students (87.7%) is planning for higher education, preferably for a Master's in Nursing or Doctorate in Nursing. These findings are consistent with the results of an Indian study, where about 88% are planning for higher education.^14^ Further, our study results revealed that about 21.1% nursing students have a plan to change the nursing profession due to reasons of lack of respect (52%), not interested (19.7%), health issue (14.5%) etc, which are consistent with study results where 17% only want to change the profession.^14^ This can be due to better opportunities of job opportunities economically and humanitarian too.

The current study revealed, there is a significant association between age and gender with low levels of perception regarding the nursing profession, indicating that younger and male student nurses were more likely to have low perception levels. These study findings were in contrast with other studies, where there was no significant association with any of the socio-demographic variables.^15^ This can be due to variation in cultural context, or nursing is predominantly a female-oriented profession.^16^ Further, the correlation analysis showed that there was a weak negative correlation identified between age and perception score, suggesting that with an increase in age, the perception becomes low towards the nursing profession. Similar results were identified in other studies, which is because of unmet expectations of the profession and burnout with time.^17^

In line with further linear regression analysis was executed and identified that gender (B=1.41, *p*=0.007) was a positive predictor, supporting that female senior nursing students tend to have a higher perception compared to males. The regression model accounted for only 3.3% (Adjusted R² = 0.033). No such study identifies this, and need for further exploration into contextual, educational and psychosocial factors that share nursing students’ perception and career outlooks.

The findings hold implications for nursing practice and nursing research internationally. Students’ perceptions towards nursing provide valuable insight into their professional identity formation, readiness for practice, and long-term commitment to the nursing workforce. Findings can inform curriculum development, policy formulation, and targeted interventions aimed at enhancing motivation, reducing attrition, and addressing gender or age-related disparities. Integrating structured career guidance, reflective practice, and role modelling into nursing education may foster positive professional outlooks and support workforce sustainability in India’s healthcare system.

## References

[1] Boniol M, Kunjumen T, Nair TS, Siyam A, Campbell J, Diallo K (2022). The global health workforce stock and distribution in 2020 and 2030: a threat to equity and ‘universal’ health coverage?. BMJ Global Health.

[2] Sharma SK, Rani R (2020). Nurse-to-patient ratio and nurse staffing norms for hospitals in India: A critical analysis of national benchmarks. Journal of Family Medicine and Primary Care.

[3] World Health Organization (2021). Global strategic directions for nursing and midwifery 2021-2025. JAMA.

[4] Roy D (2022). State of nursing in India: Persistent systemic challenges. The Lancet Regional Health - Southeast Asia.

[5] Mulisa D, Tolossa T, Oluma Ayana A, Regasa MT, Bayisa L, Abera T (2022). Nurses are leaving the nursing profession: A finding from the willingness of the nurses to stay in the nursing profession among nurses working in selected public hospitals of Wollega Zones, Oromia, Ethiopia. SAGE Open Medicine.

[6] Neumbe IM, Ssenyonga L, Soita DJ, Iramiot JS, Nekaka R (2023). Attitudes and perceptions of undergraduate nursing students towards the nursing profession. PLoS One.

[7] MsMM James (2017). A Study to Assess the Opinion regarding Pursuing Jobs in Nursing Profession among Outgoing Student Nurses in a Selected College of Nursing in New Delhi. International Journal of Nursing & Midwifery Research.

[8] Momin MI, Karade J (2017). A Study of the Perceptions towards Nursing Profession amongB.Sc. Nursing Students Enrolled in Western Maharashtra. Asian Journal of Nursing Education and Research.

[9] (2022). Khumukcham Anupama Devi SKS. International Journal of Medical Research and Review.

[10] Humane S (2022). Attitude of Nursing Students Towards Nursing Profession: Digital Survey. International Journal of Science and Healthcare Research.

[11] Ayele Woldasemayat L, Mekonnen Zeru L, Demissie Abathun A (2022). Perception towards nursing profession and associated factors among patients at Jimma Medical Center, Ethiopia. A cross- sectional study. International Journal of Africa Nursing Sciences.

[12] Aljedaani SM (2017). Nurses’ Perceptions of Nursing as a Profession and Its Impact on Their Intention to Leave Their Career: Staff Nurses in Jeddah City. IOSR Journal of Nursing and Health Science.

[13] Mary E, Muraleedharan VR, Jayapal SK, Dash U, Rajesh M (2019). Job Intentions of Nurses Trained in Public and Private Institutions in Tamil Nadu, India: A Cross-Sectional Study. Indian Journal of Nursing Sciences.

[14] Kaur K (2023). A Study to assess Future Plans and Perception about Profession among Nursing Students. Journal of Nurse Midwifery and Maternal Health.

[15] Subba R, Subba HK, Joshi A (2021). Perception towards Male Enrolment in Nursing among High School Students at Selected Schools of Bharatpur, Chitwan. International Journal of Health Sciences Research.

[16] Prosen M (2022). Nursing students’ perception of gender-defined roles in nursing: a qualitative descriptive study. BMC Nursing.

[17] Holmberg C, Wolf A, Olsson MM, Heckemann B (2022). Nurses’ general attitudes and caregiving-specific perceptions toward the oldest-old: A nationwide survey. International Journal of Nursing Studies.

